# Genome-Wide Association Study (GWAS) to Identify Salt-Tolerance QTLs Carrying Novel Candidate Genes in Rice During Early Vegetative Stage

**DOI:** 10.1186/s12284-020-00433-0

**Published:** 2021-01-09

**Authors:** Leila Nayyeripasand, Ghasem Ali Garoosi, Asadollah Ahmadikhah

**Affiliations:** 1 Agricultural Biotechnology Department, Faculty of Agriculture, Imam Khomeini International University, Qazvin, Iran; 2 Department of Plant Sciences and Biotechnology, Faculty of Life Sciences and Biotechnology, Shahid Beheshi University, G.C. Velenjak, Tehran, Iran

**Keywords:** Genome-wide association mapping, Molecular breeding, Rice, Salinity, SNPs

## Abstract

**Background:**

Rice is considered as a salt-sensitive plant, particularly at early vegetative stage, and its production is suffered from salinity due to expansion of salt affected land in areas under cultivation. Hence, significant increase of rice productivity on salinized lands is really necessary. Today genome-wide association study (GWAS) is a method of choice for fine mapping of QTLs involved in plant responses to abiotic stresses including salinity stress at early vegetative stage. In this study using > 33,000 SNP markers we identified rice genomic regions associated to early stage salinity tolerance. Eight salinity-related traits including shoot length (SL), root length (RL), root dry weight (RDW), root fresh weight (RFW), shoot fresh weight (SFW), shoot dry weight (SDW), relative water content (RWC) and TW, and 4 derived traits including SL-R, RL-R, RDW-R and RFW-R in a diverse panel of rice were evaluated under salinity (100 mM NaCl) and normal conditions in growth chamber. Genome-wide association study (GWAS) was applied based on MLM(+Q + K) model.

**Results:**

Under stress conditions 151 trait-marker associations were identified that were scattered on 10 chromosomes of rice that arranged in 29 genomic regions. A genomic region on chromosome 1 (11.26 Mbp) was identified which co-located with a known QTL region *SalTol1* for salinity tolerance at vegetative stage. A candidate gene (*Os01g0304100*) was identified in this region which encodes a cation chloride cotransporter. Furthermore, on this chromosome two other candidate genes, *Os01g0624700* (24.95 Mbp) and *Os01g0812000* (34.51 Mbp), were identified that encode a *WRKY* transcription factor (*WRKY 12*) and a transcriptional activator of gibberellin-dependent alpha-amylase expression (*GAMyb*), respectively. Also, a narrow interval on the same chromosome (40.79–42.98 Mbp) carries 12 candidate genes, some of them were not so far reported for salinity tolerance at seedling stage. Two of more interesting genes are *Os01g0966000* and *Os01g0963000*, encoding a plasma membrane (PM) H^+^-ATPase and a peroxidase BP1 protein. A candidate gene was identified on chromosome 2 (*Os02g0730300* at 30.4 Mbp) encoding a high affinity K^+^ transporter (HAK). On chromosome 6 a DnaJ-encoding gene and pseudouridine synthase gene were identified. Two novel genes on chromosome 8 including the ABI/VP1 transcription factor and retinoblastoma-related protein (RBR), and 3 novel genes on chromosome 11 including a *Lox,* F-box and Na^+^/H^+^ antiporter, were also identified.

**Conclusion:**

Known or novel candidate genes in this research were identified that can be used for improvement of salinity tolerance in molecular breeding programmes of rice. Further study and identification of effective genes on salinity tolerance by the use of candidate gene-association analysis can help to precisely uncover the mechanisms of salinity tolerance at molecular level. A time dependent relationship between salt tolerance and expression level of candidate genes could be recognized.

## Background

Drought and salinity stresses seriously threat crop production worldwide (Munns 2011). The area of saline and salt affected land will be continuously increased mainly because of climate change (Islam et al. 2019). Rice (*Oryza sativa* L.) is one of the most important cereals as it is the source of nutrition for more than 50% of the world population (Xu et al. 2016). Rice is considered as a salt-sensitive plant and its production is suffered from salinity due to expansion of salt affected land in areas under cultivation of this crop (Islam et al. 2019). Hence, significant increase of rice productivity on salinized lands is really necessary (Al-Tamimi et al. 2016). Because of the sensitivity of rice plant at the seedling and reproductive phases, salinity stress is considered to be a major limitation for the production of rice (Maas and Hoffman 1977; Singh and Flowers 2010; Reddy et al. 2017). Thus, for improvement of salt tolerance in rice, it is better to target more sensitive growth stages to salt stress such as seedling stage (Walia et al. 2005). A lot of studies show that salinity tolerance is a complex trait which is controlled by quantitative trait loci (QTL) (Roy et al. 2011), and using QTL mapping in segregating populations many QTL regions were reported for different traits under salinity stress in rice. Prasad et al. (2000) using a DH population identified a QTL for seminal root length under salt stress on chromosome 6 which explained 18.9% of phenotypic variation. Using a RIL population and AFLP markers, Koyama et al. (2001) identified a QTL for dry mass under salt stress on chromosome 6 explaining 9.7% of phenotypic variation. They also identified 3 QTLs for Na^+^ uptake/concentration on chromosomes 1, 4 and 6, and identified 4 QTLs for K^+^ uptake/concentration on chromosomes 1, 4, 6 and 9. They also identified 2 QTLs for Na^+^:K^+^ ratio on chromosomes 1 and 4. Takehisa et al. (2004) identified 12 QTLs for shoot length under salt stress on chromosomes 1, 3 and 7 that explained 12% to 30% of phenotypic variation. Aman et al. (2007) identified 5 QTLs for salt injury on chromosomes 1, 3, 4 and 5 that explained 5.8% to 25.8% of phenotypic variation. Lee et al. (2007) identified 4 QTLs for salt tolerance in RIL population on chromosomes 1 and 3 that explained 9.1% to 27.8% of phenotypic variation. Using a BIL population (BC3F5), Kim et al. (2009) identified 1, 2 and 5 QTLs for relative seedling height, relative leaf area and relative dry weight on chromosomes 1, 6 and 7. Ul Haq et al. (2010) by using a RIL population identified 1, 2, 5, 5 and 5 QTLs for salt tolerance, shoot dry weight, shoot water content, Na^+^ concentration and Na^+^:K^+^ ratio on different chromosomes of rice. Ghomi et al. (2013) using a F_2:4_ population detected 41 QTLs for 12 physiological traits that were scattered on all rice chromosomes.

The aim of genetic mapping is to identify inherited markers related to loci that control the trait of interest, particularly complex quantitative traits. Generally, two strategies are used in the genetic mapping of traits in plants: (1) linkage mapping and (2) linkage disequilibrium (LD) mapping. Linkage mapping counts the recombination events between molecular markers and the unknown genes in a segregating population developed from crosses between 2 or more parents, whereas LD mapping or association mapping (AM) measures correlation between marker alleles and the given trait in a natural population (Rosyara and Joshi 2012). Association mapping identifies a single polymorphism within a locus or a within candidate gene that create the given phenotype. With this method one can search for genotype-phenotype correlations among unrelated individuals of a species. It has high resolution since the historical recombination events were accumulated in natural populations and collections of landraces, breeding materials and varieties. By exploiting broader genetic diversity, thus AM offers three main advantages over linkage mapping: higher mapping resolution, higher allele number and time saving in establishing a marker-trait association, and hence it is a better choice in genetic mapping and breeding programmes (Flint-Garcia et al. 2003). AM has been advocated as the method of choice for identifying loci involved in the inheritance of complex quantitative traits (Risch and Merikangas 1996; Slatkin 2008). In the last two decades, AM has been used in different plant species, and based on it a lot of QTLs have been mapped or cloned (Price 2006). However, a part of detected associations can also be the result of population structure which can create false marker-trait associations. To overcome this problem it is better to control for structure effect (Q coefficients) in the statistical model (Pritchard et al. 2000). AM is not only capable of identification and mapping of QTLs of interest, but also explains the reason for polymorphism within a gene which accounts for the difference between two phenotypes (Palaisa et al. 2003).

Association mapping has been applied to study the genetic control of quality traits, seed traits such as seed longevity (Li et al. 2014), dormancy, and seed vigor, and it was applied for quantitative traits, particularly for tolerance to biotic stresses such as sheath blight resistance, and tolerance to abiotic stresses such as cold tolerance and salinity and alkalinity tolerance (Kumar et al. 2015; Al-Tamimi et al. 2016). More recently, Frouin et al. (2018) in a GWAS research detected 27 QTLs for mild salinity-related traits that were mapped on 12 chromosomes of rice. Also, using GWAS, Naveed et al. (2018) reported 20 QTLs for 11 salinity tolerance-related traits at germination and seedling stages of rice. Because of higher sensitivity of rice plant to salinity stress at seedling stage, in this study we evaluated a panel of rice accessions from International Rice Research Institute (IRRI) at early vegetative stage under salt stress and normal conditions to finely identify genomic regions associated to salinity tolerance by dense map of SNP markers, to validate earlier candidate genes and to identify novel candidate genes affecting salinity tolerance.

## Results

### Salinity Response

The analysis of variance (ANOVA) showed that treatments (normal and salinity) had significant differences for all the studied traits except for the shoot length (SL). There was a significant difference between genotypes (G) and G × T interaction for all the studied traits (Supplementary Table S2).

Mean comparisons showed that means of shoot length (SL) and turgid weight (TW) at salinity condition were not significantly differed from normal condition (Table 1). While the mean of most traits decreased significantly under salinity stress relative to normal condition; significant loss under salinity condition was observed for RL, SFW, RFW, RDW, SDW and RWC (Table 1). In addition, frequency distribution of traits under both conditions alleviated quantitative nature (Supplementary Fig. S1a&b) which is necessary for association mapping.

**Table 1:** Descriptive statistics of different traits under normal and salt stress conditions and the effect of salt stress on the studied traits

	SL (cm)	RL (cm)	SFW (mg)	RFW (mg)	SDW (mg)	RDW (mg)	TW (%)	RWC (%)
Mean (normal)	12.04	10.50	215.12	146.97	30.84	19.65	231.28	92.05
Min (normal)	4.57	9.20	84.80	15.00	6.75	3.70	91.50	76.76
Max (normal)	25.46	31.62	351.50	362.60	45.40	39.10	418.60	96.95
Sd (normal)	2.52	4.16	41.62	59.47	5.75	6.88	44.85	5.12
Mean (salt)	12.17	7.35	205.47	130.12	27.94	17.96	228.30	88.68
Min (salt)	6.92	2.00	96.86	47.70	15.90	5.50	112.70	62.75
Max (salt)	18.14	17.70	350.59	278.00	55.66	43.50	425.80	99.41
Sd (salt)	2.04	2.10	40.82	44.59	5.91	6.11	45.51	4.99
Effect of salt stress (%)	+ 1.1^ns^	−30.0^b^	−4.5^a^	−11.5^b^	−9.4^a^	−8.6^a^	−1.3^ns^	− 3.7^a^

*ns* Non-significant effects at 5% level

^a^and ^b^ indicating significance at 5% and 1% level of probability, respectively

Correlation analysis showed that under both normal and salinity conditions there were significant correlations among most of the studied traits (Supplementary Tables S3&S4). In general, correlations among traits at both conditions alleviated similar trend and magnitude.

### GWAS Results

#### Population Structure

After elimination of the monomorphic loci and loci with minor allele frequency (MAF) below 5%, from the total of 37,867 SNP markers, 33,864 markers remained in genotyping data set for analysis. The total length of the rice chromosomes is ~ 380 Mbp, hence, in average there was one SNP per 11.2 Kbp. The highest number of markers were on chromosome 1 (5272 SNPs), and the lowest number of markers were on chromosome 9 (2111 SNPs). As seen in supplementary Fig. S2a, based on Evanno et al. (2005) method the entire population consisted from 4 sub-populations which were depicted in a bar plot in supplementary Fig. S2b.

### Identification of Associated Genomic Regions to the Studied Traits

**Fig. 1:**
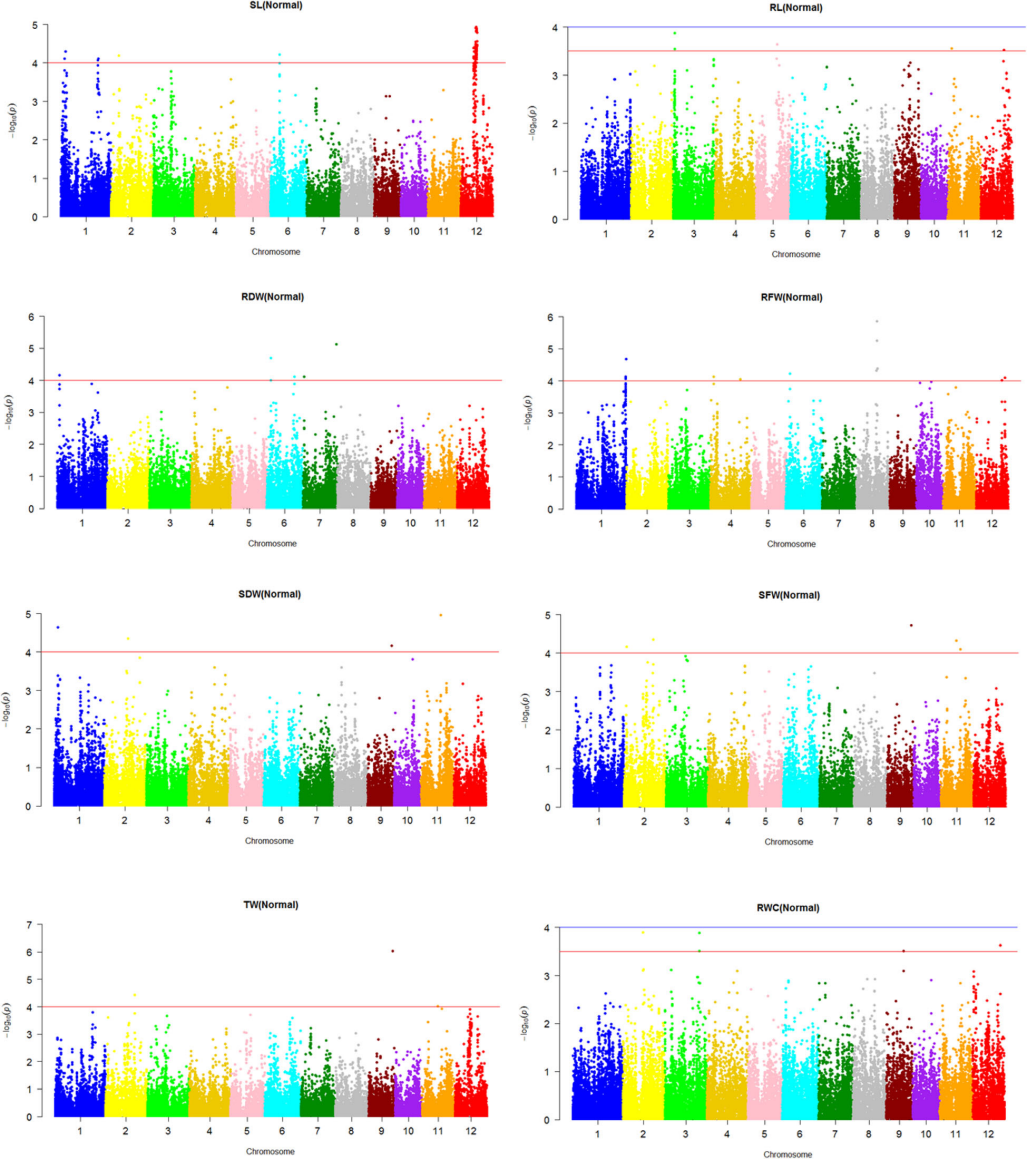
Manhattan plots of p-values analyzed using mixed linear model (MLM) controlled for population structure and kinship of rice genotypes for different traits under normal condition

**Fig. 2:**
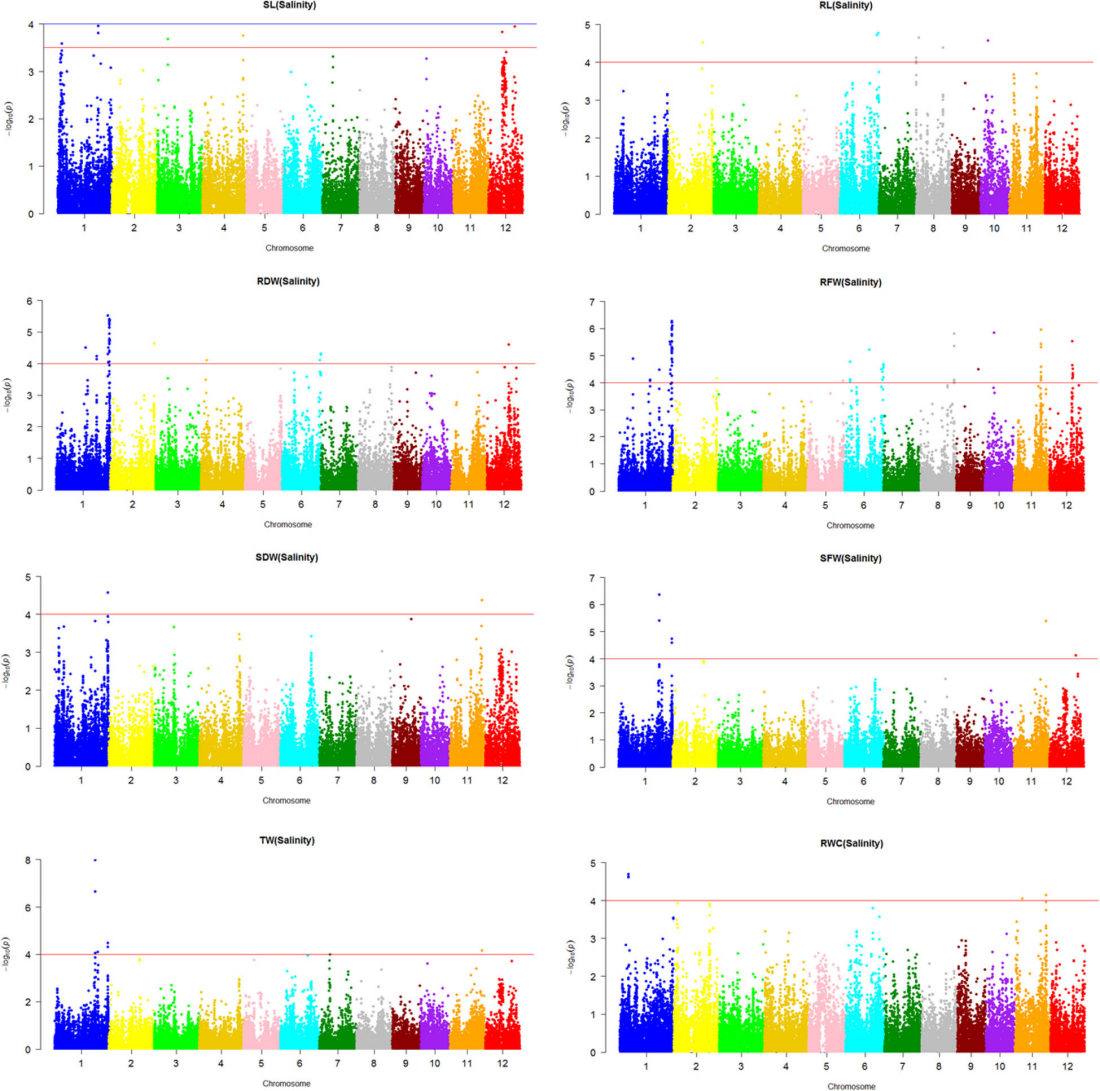
Manhattan plots of p-values analyzed using mixed linear model (MLM) controlled for population structure and kinship of rice genotypes for different traits under salinity condition

The association between the SNP marker genotypes and the tolerance-related traits was assessed by the use of a mixed linear model (MLM) controlled for the population structure (Q matrix; Supplementary Table S5) and kinship coefficient of varieties (K). Significant marker-trait associations were identified by negative log10 (*p* value) and by r^2^ as part of the variations explained by SNP markers. The association analysis between 8 evaluated traits and SNP markers was conducted at normal and salinity stress conditions and the results have been presented in the form of Manhattan plots (Figs. 1 and 2).

Under normal and stress conditions the MLM-Q-K analyses with –log_10_(p) > 4.0 identified 97 and 151 trait-marker associations, respectively (Supplementary Tables S6&S7). Chromosomes 3, 5 and 10 under normal condition, and chromosomes 3 and 7 under salinity conditions lake any marker-trait associations. In contrast, chromosome 12 under normal condition and chromosome 1 under salinity condition had maximum number of marker-trait associations.

For shoot length (SL) under normal condition, 5 genomic regions tagged to 65 SNP markers on chromosomes 1, 2, 6 and 12 showed a significant association (Fig. 1, Table 2). However, under salinity stress, the association analysis identified 2 genomic regions tagged to 2 SNPs on the chromosomes 1 and 12 (Fig. 2). These markers explained 9.26 to 10.14% of phenotype variation of the trait. The strongest QTL placed on chromosome 1 at position 32.48 Mbp (Table 2).

**Table 2:** Locations and characteristics of QTLs detected for shoot length (SL) under normal and salinity conditions

QTL	Chromosome	No. of Associated SNPs	Position/interval (Mbp)	-Log_10_(p)	r^2^
qSLn1.1	1	2	3.40–4.01	4.1–4.30	10.16–10.30
qSLn1.2	1	2	31.80–32.48	4.1	10.15–10.74
qSLn2.1	2	1	6.62	4.2	12.23
qSLn6.1	6	1	8.19	4.2	10.19
qSLn12.1	12	59	11.06–14.22	4.0–4.9	9.64–14.12
qSLs1.1	1	1	32.48	4.00	10.14
qSLs12.1	12	1	21.08	4.00	9.26

For root length (RL) under normal condition no SNP marker showed significant association. However, under salinity stress 6 genomic regions were identified with a significant association with RL (Fig. 2). Eight SNP markers on chromosomes 2, 6, 8 and 10 showed significant associations which explained 7.98 to 14.75% of phenotype variation of the trait. The strongest QTL placed on chromosome 6 at position 30.92 Mbp (Table 3).

**Table 3:** Locations and characteristics of QTLs detected for roots length (RL) under normal and salinity conditions

QTL	Chromosome	No. of Associated SNPs	Position/interval (Mbp)	-Log_10_(p)	r^2^
qRLs2.1	2	1	27.27	4.5	10.92
qRLs6.1	6	2	29.76–30.92	4.7–4.8	10.75–14.75
qRLs8.1	8	2	0.06–0.12	4.0–4.1	9.12–10.11
qRLs8.2	8	1	2.33	4.7	10.58
qRLs8.3	8	1	21.51	4.4	10.22
qRLs10.1	10	1	6.12	4.6	11.89

In the case of root dry weight (RDW) under normal condition, 5 genomic regions tagged to 6 SNP markers on the chromosomes 1, 6 and 7 showed a significant association (Fig. 1). These markers explained 9.77 to 11.27% of phenotype variation of the trait (Table 4). However, under salinity stress condition, 7 genomic regions tagged to 39 SNP markers were identified which had a significant association with RDW (Fig. 2). These SNP markers were localized on chromosomes 1, 2, 4, 6 and 12 which explained 7.60 to 14.38% of phenotype variation of the trait. The strongest QTL was on chromosome 1 at position 41.35 Mbp (Table 4).

**Table 4:** Locations and characteristics of QTLs detected for root dry weight (RDW) under normal and salinity conditions

QTL	Chromosome	No. of Associated SNPs	Position/interval (Mbp)	-Log_10_(p)	r^2^
qRDWn1.1	1	1	2.06	4.2	10.04
qRDWn6.1	6	2	3.59–3.60	4.0–4.7	10.12–11.27
qRDWn6.2	6	1	23.70	4.1	9.77
qRDWn7.1	7	1	0.92	4.1	9.82
qRDWn7.2	7	1	28.93	5.1	10.96
qRDWs1.1	1	1	23.67	4.5	7.99
qRDWs1.2	1	2	32.33–32.36	4.1–4.2	9.31–9.49
qRDWs1.3	1	30	41.12–42.98	4.0–5.4	9.57–14.37
qRDWs2.1	2	1	35.26	4.7	7.60
qRDWs4.1	4	1	4.69	4.1	8.46
qRDWs6.1	6	3	30.48–31.21	4.1–4.3	9.23–9.91
qRDWs12.1	12	1	17.53	4.6	11.26

For root fresh weight (RFW) under normal condition, 7 genomic regions tagged to 14 SNPs showed a significant association (Fig. 1). The markers were localized on chromosomes 1, 4, 6, 8 and 12, and explained 6.62 to 11.63% of phenotype variation of the trait (Table 5). However, under the salinity stress condition the association analysis identified 15 genomic regions having a significant association with the root fresh weight (RFW) (Fig. 2). Sixty eight SNP markers on the chromosomes 1, 2, 5, 6, 8, 9, 10, 11 and 12 showed significant associations with the trait. These markers explained 5.58 to 14.62% of phenotype variation of the trait. The strongest QTL placed on chromosome 12 at position 18.46 Mbp (Table 5).

**Table 5:** Locations and characteristics of QTLs detected for roots fresh weight (RFW) under normal and salinity conditions

QTL	Chromosome	No. of Associated SNPs	Position/interval (Mbp)	-Log_10_(p)	r^2^
qRFWn1.1	1	5	42.44–42.98	4.1–4.7	6.71–7.77
qRFWn4.1	4	1	2.52	4.14	7.04
qRFWn4.2	4	1	25.86	4.1	6.62
qRFWn6.1	6	1	3.59	4.2	7.18
qRFWn8.1	8	4	16.97–180.89	4.2–5.9	7.18–11.63
qRFWn12.1	12	1	22.14	4.0	6.92
qRFWn12.2	12	1	24.84	4.1	6.71
qRFWs1.1	1	1	11.26	4.9	10.11
qRFWs1.2	1	2	24.91–24.95	4.1	8.10–8.11
qRFWs1.3	1	1	32.33	4.5	6.39
qRFWs1.4	1	34	40.79–42.98	4.3–6.3	6.60–13.16
qRFWs2.1	2	1	35.26	4.2	5.93
qRFWs5.1	5	2	28.54	4.1	5.58
qRFWs6.1	6	3	4.58–4.61	4.1–4.8	5.67–6.90
qRFWs6.2	6	1	20.04	5.2	11.48
qRFWs6.3	6	10	29.76–31.21	4.1–4.7	5.77–6.76
qRFWs8.1	8	9	26.72–26.94	4.0–5.8	5.63–9.25
qRFWs9.1	9	1	17.96	4.5	9.10
qRFWs10.1	10	1	7.32	5.8	9.15
qRFWs11.1	11	8	21.68–21.98	4.1–6.0	6.06–14.61
qRFWs12.1	12	8	18.46–18.60	4.2–5.5	9.96–14.62

For shoot fresh weight (SFW) under normal condition, 5 genomic regions tagged to 5 SNP markers on chromosomes 2, 9 and 11 showed a significant association (Fig. 1). These markers explained 10.17 to 12.25% of phenotype variation of the trait (Table 6). However, under salt stress 4 genomic regions tagged to 6 SNP markers on chromosomes 1, 11 and 12 showed a significant association with SFW (Fig. 2). These markers explained 9.90 to 16.93% of phenotype variation of the trait. The strongest QTL placed on chromosome 1 at position 32.48 Mbp (Table 6).

**Table 6:** Locations and characteristics of QTLs detected for shoot fresh weight (SFW) under normal and salinity conditions

QTL	Chromosome	No. of Associated SNPs	Position/interval (Mbp)	-Log_10_(p)	r^2^
qSFWn2.1	2	1	2.13	4.2	10.17
qSFWn2.2	2	1	25.25	4.3	10.53
qSFWn9.1	9	1	20.94	4.7	12.25
qSFWn11.1	11	1	13.86	4.3	11.30
qSFWn11.2	11	1	17.02	4.09	11.10
qSFWs1.1	1	2	32.48–32.54	5.4–6.4	13.38–16.93
qSFWs1.2	1	2	42.43–42.44	4.6–4.8	11.51–12.34
qSFWs11.1	11	1	25.85	5.4	13.16
qSFWs12.1	12	1	21.08	4.1	9.90

For shoot dry weight (SDW) under normal condition, 4 genomic regions tagged to 4 SNP markers on chromosomes 1, 2, 9 and 11 showed a significant association (Fig. 1). These markers explained 10.60 to 13.66% of phenotype variation of this trait (Table 7). However, under salinity stress 2 genomic regions tagged to 2 SNP markers on chromosomes 1 and 11 were identified with a significant association with shoot dry weight (SDW) (Fig. 2). These markers explained 10.59 to 11.13% of phenotype variation of this trait. The strongest QTL placed on chromosome 1 at position 42.43 Mbp (Table 7).

**Table 7:** Locations and characteristics of QTLs detected for shoot dry weight (SDW) under normal and salinity conditions

QTL	Chromosome	No. of Associated SNPs	Position/interval (Mbp)	-Log_10_(p)	r^2^
qSDWn1.1	1	1	33.98	4.6	12.22
qSDWn2.1	2	1	20.35	4.4	10.60
qSDWn9.1	9	1	20.94	4.2	10.74
qSDWn11.1	11	1	17.02	5.0	13.67
qSDWs1.1	1	1	42.43	4.6	11.13
qSDWs11.1	11	1	25.85	4.4	10.59

For relative water content (RWC) under normal condition, no SNP marker was associated. These markers explained 8.36 to 10.08% of phenotype variation of this trait (Table 8). However, under salinity stress condition 3 genomic regions tagged to 5 SNP markers on chromosomes 1 and 11 were identified which were associated to RWC (Fig. 2). These markers explained 10.07 to 11.64% of phenotype variation of the trait. The strongest QTL placed on chromosome 1 at position 7.09 Mbp (Table 8).

**Table 8:** Locations and characteristics of QTLs detected for relative water content (RWC) under normal and salinity conditions

QTL	Chromosome	No. of Associated SNPs	Position/interval (Mbp)	-Log_10_(p)	r^2^
qRWCs1.1	1	3	7.09	4.6–4.7	11.28–11.64
qRWCs11.1	11	1	6.06	4.1	11.16
qRWCs11.2	11	1	24.96	4.2	10.07

**Fig. 3:**
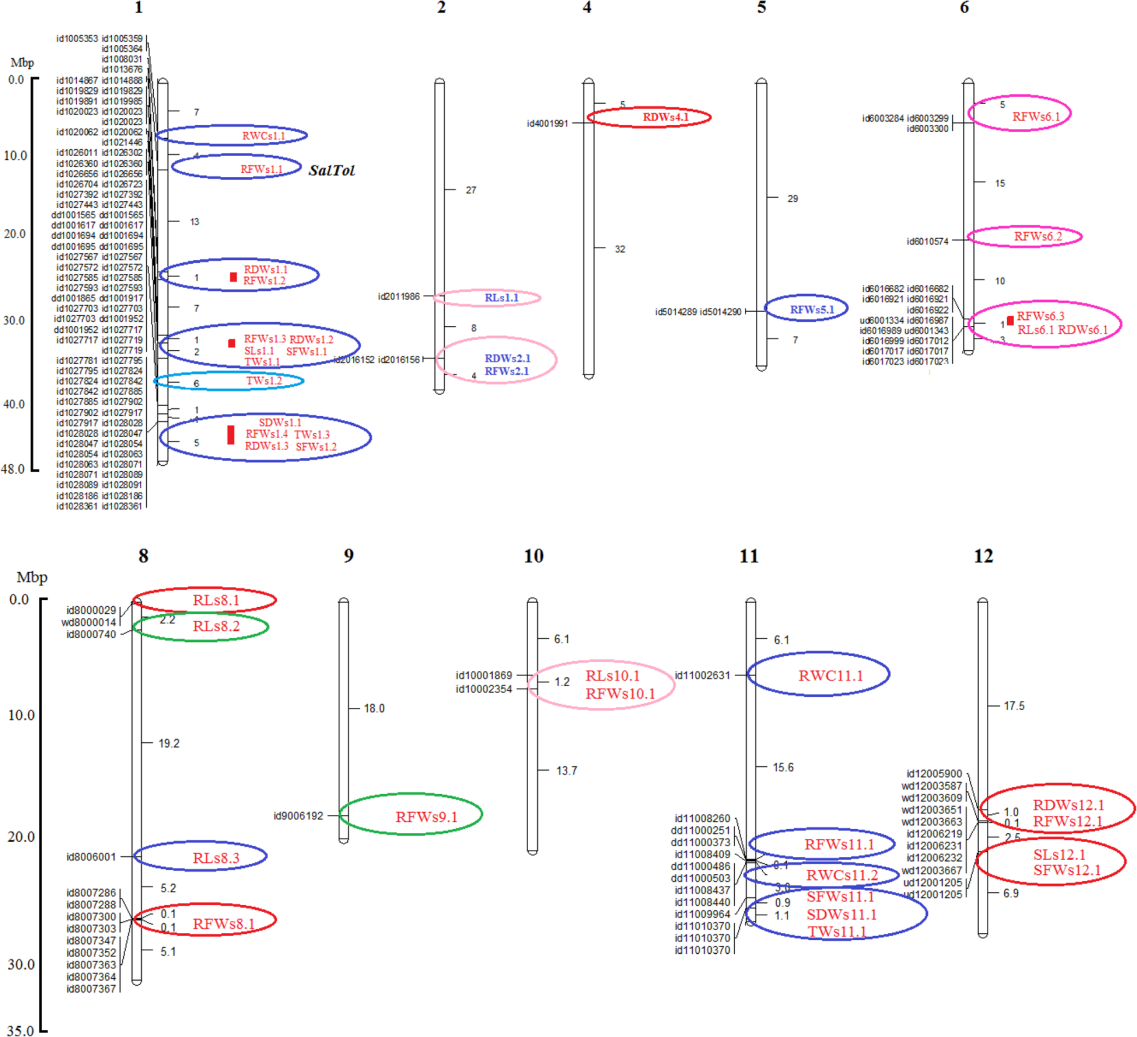
Distribution of GWAS-based detected QTLs on 10 chromosomes of rice. Distances on the map are in Mbp. The position of *SalTol*, a well-studied QTL at seedling stage, was shown on chromosome 1

For turgid weight (TW) under normal condition, 3 genomic regions tagged to 3 SNP markers on the chromosomes 2, 9 and 11 showed a significant association (Fig. 1). These markers explained 10.48 to 15.83% of phenotype variation of this trait (Table 9). However, under salt stress condition 4 genomic regions tagged to 7 SNP markers showed a significant association to TW (Fig. 2). The markers were located on chromosomes 1 and 11 which explained 9.82 to 21.17% of phenotype variation of the trait. The strongest QTL placed on chromosome 1 at position 32.48 Mbp (Table 9). All detected QTLs are illustrated on the rice chromosomal map (Fig. 3).

**Table 9:** Locations and characteristics of QTLs detected for turgid weight (TW) under normal and salinity conditions

QTL	Chromosome	No. of Associated SNPs	Position/interval (Mbp)	-Log_10_(p)	r^2^
qTWn2.1	2	1	25.25	4.4	10.72
qTWn9.1	9	1	20.94	6.0	15.83
qTWn11.1	11	1	13.86	4.0	10.48
qTWs1.1	1	3	32.46–32.54	4.1–8.0	9.95–21.17
qTWs1.2	1	1	34.51	4.1	9.82
qTWs1.3	1	2	42.43–42.44	4.3–4.5	10.38–12.02
qTWs11.1	11	1	25.85	4.2	9.99

For other derived traits including (SL-R, RL-R, RFW-R and RDW-R) 12 genomic regions tagged to 48 SNP markers on different chromosomes showed significant associations (supplementary Fig. S3). For SL-R two genomic regions on chromosomes 2 and 8 (at intervals of 30.42–30.55 and 14.06–14.99 Mbp, respectively) were identified that explained 9.37 to 16.04% of phenotypic variation of the trait. For RL-R three genomic regions on chromosomes 2, 7 and 9 were identified that explained 9.15 to 16.46% of phenotypic variation of the trait. For RFW-R four genomic regions on chromosomes 1, 2, 5 and 10 were identified that explained 9.84 to 18.64% of phenotypic variation of the trait. For RDW-R three genomic regions on chromosomes 1, 2 and 7 were identified that explained 10.06 to 18.05% of phenotypic variation of the trait (Table 10).

**Table 10:** Locations and characteristics of QTLs detected for other relative traits (including SL-R, RL-R, RFW-R and RDW-R)

QTL	Chromosome	No. of Associated SNPs	Position/interval (Mbp)	-Log_10_(p)	r^2^
qSL-R2.1	2	5	30.42–30.55	4.0–5.3	11.11–12.93
qSL-R8.1	8	4	14.06–14.99	4.0–6.0	9.37–16.04
qRL-R2.1	2	2	34.98–35.01	4.1–5.5	9.73–13.07
qRL-R7.1	7	1	19.83	5.5	16.46
qRL-R9.1	9	2	16.29–16.41	4.0–4.1	9.15–9.50
qRFW-R1.1	1	7	22.77–22.99	4.1–5.2	9.95–12.89
qRFW-R2.1	2	7	30.42–30.55	4.1–7.5	9.84–18.64
qRFW-R5.1	5	2	18.69–18.70	4.9–5.2	11.89–12.43
qRFW-R10.1	10	4	17.08–17.13	4.3–5.4	10.35–15.23
qRDW-R1.1	1	5	22.77–22.84	4.2–4.3	10.06–11.38
qRDW-R2.1	2	5	30.42–30.55	4.3–5.7	10.32–13.90
qRDW-R7.1	7	3	19.66–19.67	4.5–7.2	10.90–18.05

### Co-Location of QTLs Under Salinity Condition

Investigation of co-location of the detected QTLs under salinity condition showed that 9 genomic regions harbored co-located QTLs (Fig. 3, Table 11). Most complex regions are two genomic regions on chromosome 1 each having 5 co-located QTLs; first one at a narrow interval of 32.33–32.54 Mbp comprised from qRDWs1.2, qRFWs1.3, qSLs1.1, qSFWs1.1, qTWs1.1, and second one at an adjacent region at 40.79–42.98 Mbp interval comprised from qRDWs1.3, qRFWs1.4, qSFWs1.2, qSDWs1.1, qTWs1.3. A genomic region on chromosome 11 also harbors 4 co-located QTLs at 24.96–25.85 Mbp interval including QTLs of qSDWs11.1, qSFWs11.1, qTWs11.1, qRWCs11.2. Furthermore, a genomic region of chromosome 6 at 29.76–31.21 Mbp interval carries 3 co-located QTLs including qRDWs6.1, qRFWs6.3, qRLs6.1. Eight other genomic regions on chromosomes 1, 2, 7, 10 and 12 carry a minimum number of co-located QTLs (e.g. two QTLs at each interval).

**Table 11:** Co-located QTLs associated to different traits under salinity stress

#	Chromosome	Position/interval (Mbp)	No. of co-located QTLs	Co-located QTLs
1	1	22.77–22.99	2	qRFW-R1.1, qRDW-R1.1
2	1	23.67–24.95	2	qRDWs1.1, qRFWs1.2
3	1	32.33–32.54	5	qRDWs1.2, qRFWs1.3, qSLs1.1, qSFWs1.1, qTWs1.1
4	1	40.79–42.98	5	qRDWs1.3, qRFWs1.4, qSFWs1.2, qSDWs1.1, qTWs1.3
5	2	30.42–30.55	2	qRFW-R2.1, qRDW-R2.1
6	2	35.26	2	qRDWs2.1, qRFWs2.1
7	6	29.76–31.21	3	qRDWs6.1, qRFWs6.3, qRLs6.1
8	7	19.66–19.83	2	qRL-R7.1, qRDW-R7.1
9	10	6.12–7.32	2	qRFWs10.1, qRLs10.1
10	11	24.96–25.85	4	qSDWs11.1, qSFWs11.1, qTWs11.1, qRWCs11.2
11	12	17.53–18.60	2	qRDWs12.1, qRFWs12.1
12	12	21.08	2	qSFWs12.1, qSLs12.1

### Identification of Candidate Genes for Salinity Tolerance

**Fig. 4:**
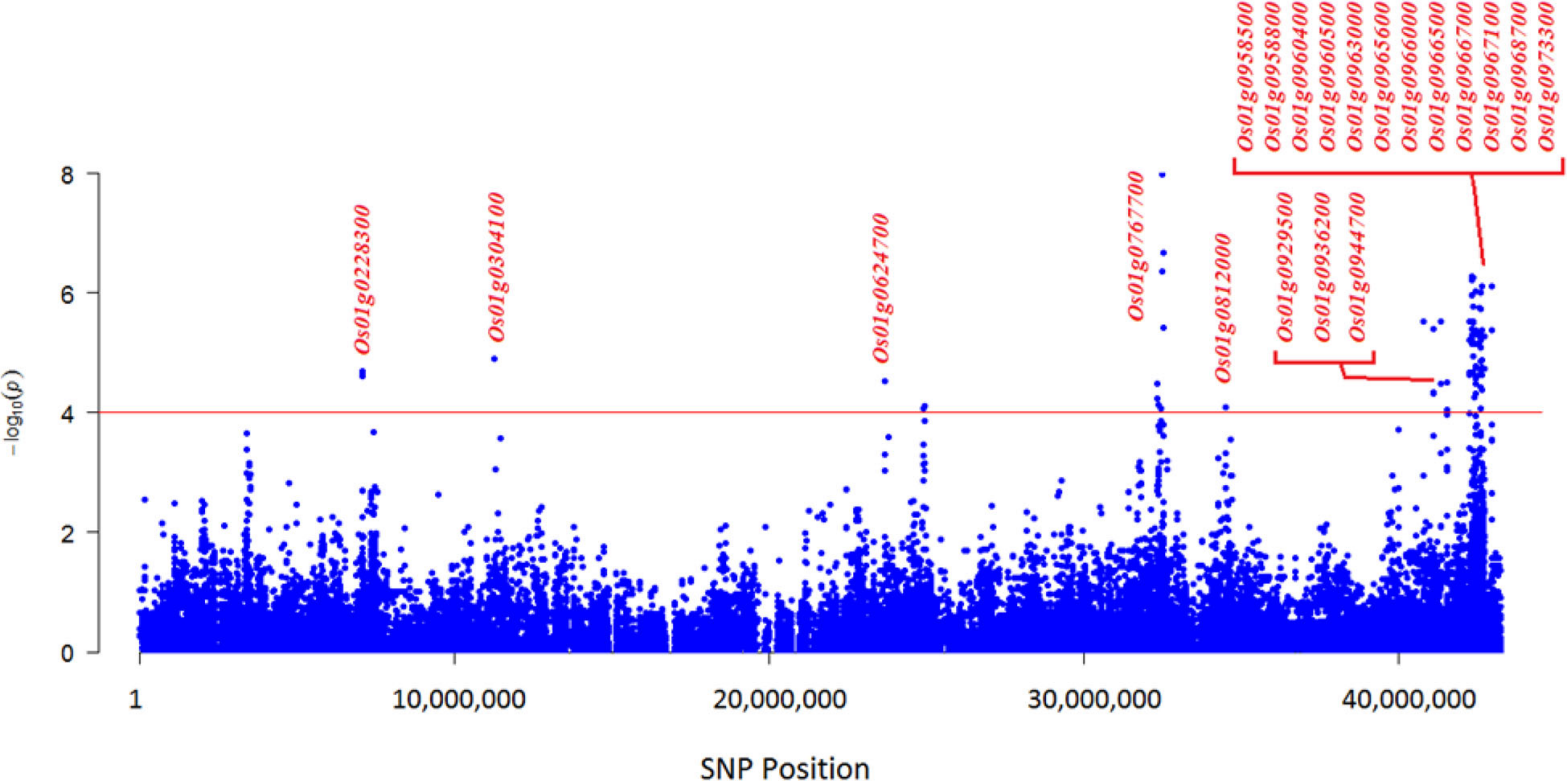
Map location of salinity-tolerance candidate genes on 7 distinct regions of chromosome 1. Gene RAP locus IDs are in red

Comparison of the physical location of SNP markers that showed significant associations to studied traits in this study, with the physical location of rice genes deposited on the Rice Annotation Project database (https://rapdb.dna.affrc.go.jp/), resulted in the identification of 36 candidate genes that may be responsible to salinity tolerance (Table 12), such that among the significant markers related to one or more traits under the salinity stress, we found many SNP markers which physically placed within the gene sequences of RAP ID database. The results showed that these genes are distributed on different genomic regions of 5 chromosomes of rice. Twenty candidate genes were identified on chromosome 1 (Table 12; Fig. 4), 2 of which are uniquely related to root dry weight (RDW), 4 genes are related to root fresh weight (RFW), one gene is related to turgid weight (TW), one gene is related to relative water content (RWC) and 12 genes are related to both RDW and RFW. The latter 12 genes scattered between a coordinate of 42.25–42.98 Mbp which coincides to 4th co-located QTL region (defined in Table 11) carrying 5 QTLs of qRDWs1.3, qRFWs1.4, qSFWs1.2, qSDWs1.1, qTWs1.3.

**Table 12:** Candidate genes in genomic regions associated to salinity tolerance. Details are given for RAP locus IDs and their putative functions from where the respective SNP was selected. Last column shows the tissue in which the given gene has highest transcription at normal condition based on Unigene database (supplementary Table S8)

Chromosome	Related traits	Position of associated SNP (bp)	Gene RAP ID	Gene function	Tissue with highest transcription
1	RWC	7,086,172	*Os01g0228300*	Mpv17/PMP22 family protein	Leaf
	RFW	11,259,217	*Os01g0304100*	Cation chloride cotransporter	Seed
	RFW	24,946,338	*Os01g0624700*	WRKY transcription factor 12	–
	RDW	32,358,779	*Os01g0767700*	DEIH-box RNA/DNA helicase	Panicle
	TW	34,510,717	*Os01g0812000*	Transcriptional activator of gibberellin-dependent alpha-amylase expression (*GAMyb*)	Seed
	RFW	40,794,206	*Os01g0929500*	Carbonyl reductase-like protein	Panicle
	RFW	41,101,759	*Os01g0936200*	Lipase class 3 family protein	Stem
	RDW	41,534,764	*Os01g0944700*	Beta-1-glucanase precursor	Stem
	RDW, RFW	42,254,208	*Os01g0958500*	RNA binding protein-like	Leaf
	RDW, RFW	42,267,480	*Os01g0958800*	Protein of unknown function DUF2305 domain containing protein	Flower
	RDW, RFW	42,339,521	*Os01g0960400*	Protein kinase core domain containing protein	Stem
	RDW, RFW	42,351,049	*Os01g0960500*	Zinc finger RING/FYVE/PHD-type domain containing protein	Seed
	RDW, RFW	42,442,204	*Os01g0963000*	Peroxidase BP 1 precursor	Leaf
	RDW, RFW	42,575,260	*Os01g0965600*	Growth inhibition and differentiation-related protein 88	Root
	RDW, RFW	42,591,390	*Os01g0966000*	Plasma membrane H^+^-ATPase (EC 3.6.1.3)	Stem/leaf
	RDW, RFW	42,622,164	*Os01g0966500*	Vacuolar protein sorting protein 55	Vegetative meristem
	RDW, RFW	42,627,050	*Os01g0966700*	Beta-fructofuranosidase (EC 3.2.1.26)	Leaf
	RDW, RFW	42,649,896	*Os01g0967100*	Ascorbate peroxidase 2	Seed
	RDW, RFW	42,723,932	*Os01g0968700*	tRNA isopentenyltransferase family protein	Vegetative meristem
	RDW, RFW	42,976,353	*Os01g0973300*	Armadillo-like helical domain containing protein, Adaptin	Flower
2	SL-R, RDW-R	30,423,080	*Os02g0730300*	High-affinity Potassium(K^+^) Transporter 25, *OsHAK25*	Stem
5	RFW	28,534,662	*Os05g0572900*	Pentatricopeptide repeat domain containing protein	Flower
6	RFW	4,612,802	*Os06g0191200*	Zinc ion binding	Flower
	RFW	20,037,449	*Os06g0535300*	DnaJ domain protein C55	Leaf
	RFW, RL	29,757,694	*Os06g0704600*	Delta-aminolevulinic acid dehydratase	Leaf
	RFW	30,484,421	*Os06g0717400*	Pseudouridine synthase domain containing protein	Stem
	RFW	31,018,842	*Os06g0728600*	EPS15 homology (EH) domain containing protein	Vegetative meristem
	RFW	31,144,213	*Os06g0730600*	Peptidase S9A prolyl oligopeptidase family protein	Seed
8	RL	63,899	*Os08g0101000*	ABI3/VP1 transcription factor family protein	Flower
	RL	125,422	*Os08g0102250*	H0913C04.5 protein	–
	RFW	26,726,527	*Os08g0535200*	MtN3-like protein; OsXa13	Panicle
	RFW	26,752,345	*Os08g0535700*	Glycerophosphodiester phosphodiesterase	Seed
	RFW	26,906,046	*Os08g0538200*	Protein of unknown function DUF247 plant family protein	–
	RFW	26,926,958	*Os08g0538700*	Retinoblastoma-related protein	Callus
11	RWC	6,061,643	*Os11g0216000*	Pyruvate kinase family protein	Flower
	RFW	21,681,513	*Os11g0575600*	Lipoxygenase 10	Callus
	RFW	21,851,184	*Os11g0578700*	F-box domain containing protein	Callus

Furthermore, one candidate gene (*Os05g0572900*) on chromosome 5 was identified under salinity stress condition which showed a significant association to root fresh weight (RFW). Six candidate genes were identified on chromosome 6 under salinity stress condition (Table 12), one of which had a significant association to two traits including root fresh weight (RFW) and root length (RL), and 5 ones had unique associations to root fresh weight (RFW). Six candidate genes were identified on chromosome 8 under salinity stress, 2 of which had a significant association to root length (RL), and 4 other genes had a significant association to RFW. One and two candidate genes were identified on chromosome 11 with a significant association to RWC and RFW, respectively (Table 12).

### Haplotype Analysis

Haplotype analysis revealed 2 distinct blocks on rice genome: first block on chromosome 1 spanning 42.25 to 42.65 Mbp which carry 7 candidate genes, and 2nd block on chromosome 8 spanning 26.72 to 26.93 Mbp which carry 3 candidate genes (supplementary Table S9). Two major haplotypes were identified within first block on chromosome 1; superior haplotype (with frequency of 90.2%) decreased both RFW and RDW, but inferior haplotype (with frequency of 9.1%) increased values of the 2 traits; so that average RFW and RDW of haplotype 1 was 125.1 and 17.3 mg/plant, respectively, while average of RFW and RDW of haplotype 2 was 180.0 and 24.4 mg/plant, respectively. In the case of 2nd block, 4 different haplotypes were identified with frequencies of 44.8, 22.7, 16.9 and 14.9%. Haplotype 4 had highest values for RFW and RDW (166.3 and 22.0 mg/plant) and haplotype 3 had lowest values of these traits (93.8 and 13.4 mg/plant). Superior haplotype (haplotype 1 with frequency of 44.8%) also produced relatively high values of RFW and RDW (142.9 and 19.6 mg/plant, respectively).

### Expression Assay of Candidate Genes

Network analysis using riceFREND database revealed co-expression pattern of the candidate genes. For each candidate gene we detected 2 to 6 direct interactions in gene networks (supplementary Table S10). For example, in the case of *Os01g0963000* (Peroxidase BP1 precursor) co-expressed with four genes including *Os03g0225900* (Allene oxide synthase), *Os06g0521500* (Haem peroxidase family protein), *Os07g0542400* (Similar to Receptor protein kinase) and *Os08g0508800* (Lipoxygenase, chloroplast precursor). *Os01g0966000* (encoding a Plasma membrane H^+^-ATPase) co-expressed with *Os03g0591000*, *Os08g0326100*, *Os11g0661000* and *Os12g0613200*.

**Fig. 5:**
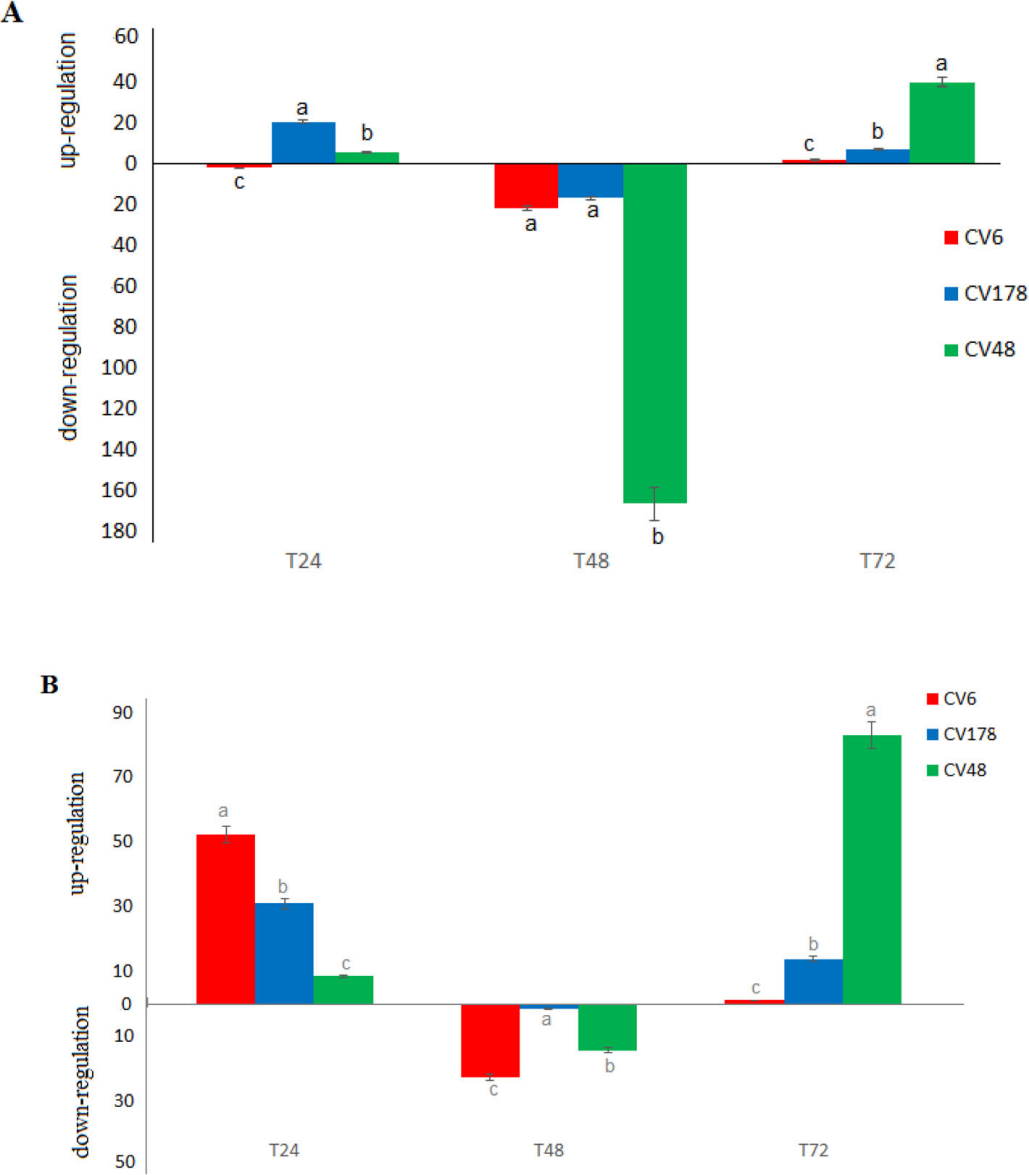
Changes in relative gene expression level of candidate genes at different times after salinity stress in different rice cultivars. **a** *Peroxidase BP1*, **b** *PM H*^*+*^*-ATPase*. T24, T48 and T72: 24, 48 and 72 h after salt stress. CV6 and CV178 (ARC6578 and Shoemed): sensitive cultivars. CV48 (Bombilla): tolerant cultivar

For validation of the response to salinity of candidate genes, an expression assay was conducted under normal and salinity conditions using sensitive and tolerant accessions. The expression of *PM H*^*+*^*-ATPase* (*Os01g0966000*) at early times after stress was increased in both tolerant and sensitive cultivars but it decreased after 72 h in sensitive cultivar and increased in tolerant cultivar (Fig. 5). The expression of *Peroxidase BP1 precursor* (*Os01g0963000*) was increased up to 40 times at 72 h after stress in tolerant cultivar.

## Discussion

Association study as an efficient method identifies the genomic regions controlling quantitative traits on the basis of linkage disequilibrium (LD) (Gupta et al. 2005). The amount of LD on the whole genome and chromosomes might significantly influence the mapping ability and selection efficacy by the help of the associated markers. In this study, the amount of LD on the basis of r^2^ values was variable from 0.254 within chromosome 11 to 0.395 within chromosome 3 with the mean value of 0.321 (data not shown). Altogether, 70.37% of the SNP markers showed significant LD (*p* < 0.01). In the association mapping method, the resolution and accuracy of the obtained map depend on the extent and amount of LD decline (Remington et al. 2001; Kim et al. 2007; Pritchard et al. 2000b; Bastien et al. 2014a, b). In another hand, LD measure in a mapping population presents an estimation of the number of required markers for the identification of QTLs (Remington et al. 2001; Kim et al. 2007; Pritchard et al. 2000; Bastien et al. 2014a, b). In the study of the core collection of rice by the use of SSR markers, it has been reported that 46.8% of the markers had significant LD (Zhang et al. 2011) which is lower than that of our study. In contrast, in the study of Jin et al. (2010) in a rice panel of 416 rice genotypes (including wild types, cultivar and breeding lines), 63% of the markers showed significant LD, which is comparable to LD obtained in our study. Zhang et al. (2011) stated that the low level of LD leads to unsuccessful scanning of the whole genome; and as a result, there would be a need for a higher number of markers. Considering the high and appropriate level of LD in our study (70.37%), it seems that the number of our SNP markers was enough to conduct association mapping.

As we showed, with a high density of SNP markers (1 SNP per 11 Kbp), the genome-wide association study (GWAS) method could facilitate fine mapping and identification of the genomic regions that control salinity tolerance-related traits at early vegetative stage. In our GWAS, many genomic regions were identified which were related to different traits under salt stress condition. Ninety seven and 151 marker-trait associations were identified by MLM-Q-K model under normal and salinity stress conditions, respectively. Kumar et al. (2015) also detected more association signals under salinity stress than under normal condition in their GWAS analysis using 4191 SNPs. The 151 associations signals under salinity stress were scattered on 10 chromosomes of rice (all chromosomes except for chromosomes 3 and 7), and altogether they were arranged in 29 genomic regions, 9 of which harbored 2 to 5 QTLs, and each of the remained 20 genomic regions comprised of only one QTL (Fig. 3). For eight studied traits viz. SL, RL, RDW, RFW, SFW, SDW, RWC and TW we identified 2, 6, 7, 15, 4, 2, 3 and 4 genomic regions under salinity condition.

The position of qRWCs1.1 on chromosome 1 (7.09 Mbp) is comparable to QTL #2138 for salt injury reported by Amman et al. (2007), and also it is comparable to q01_03 for SDW and RDW reported by Frouin et al. (2018) (supplementary Table S11). The position of qRFWs1.1 on the same chromosome (11.26 Mbp) is similar to that of QTLs #2181, #2182 and #2183 (for salt tolerance, shoot dry weight and leaf water content, respectively) reported by Ul Haq et al. (2010); also a QTL for leaf area (q01_04) was mapped in this region by Frouin et al. (2018). This region also co-locates with qSKC-1, known as *SalTol1*, a major QTL which controls salinity tolerance at vegetative stage of rice (Lin et al. 2004). Pandit et al. (2010) also mapped 3 co-located QTLs for salinity tolerance in the same genomic region (including qNaSH-1.1, qKSH-1.1 and qNa/KSH-1.1) using a RIL population from a cross between CSR27 and MI48. The positions of QTLs qRDWs1.1 and qRFWs1.2 on chromosome 1 (23.67 and 24.91–24.95 Mbp, respectively) are comparable to q01_5 and q01_6 for tiller number and specific leaf area reported by Frouin et al. (2018) and to qSNC1 for shoot Na^+^ concentration detected in a GWAS analysis by Naveed et al. (2018).

Among 8 genomic regions associated to different traits on chromosome 1, two genomic regions represented more importance for salinity tolerance; these are located in a coordinate of 32.33–34.51 Mbp interval showing association to SL, RDW, RFW, SFW and TW, and in a coordinate of 40.79–42.98 Mbp interval showing association to RDW, RFW, SFW, SDW and TW. Out of these, the location of QTL identified for turgid weight within this region (qTWs1.1 at 34.51 Mbp) is comparable to major QTL #2122 for shoot length reported by Takehisa et al. (2004). The locations of 2 QTLs of qRFWs1.4 and qRDWs1.3 on chromosome 1 (at positions of 40.79 and 41.12 Mbp, respectively) are comparable to QTLs #2123, #2124 and #2125 for shoot length under salinity reported by Takehisa et al. (2004). The QTL position also is similar to q01_09 for leaf area under salinity condition reported by Frouin et al. (2018). The location of QTL qRLs2.1 (27.27 Mbp) is comparable to 4 QTLs for tiller number detected by Takehisa et al. (2004) and to q02_10 for leaf area detected by Frouin et al. (2018). The QTL qRDWs4.1 on chromosome 4 (4.69 Mbp) co-locates to qGR4 for germination rate reported by Naveed et al. (2018), and the QTL qRFWs5.1 on chromosome 5 (28.54 Mbp) co-locates to QTL #2142 for salt injury reported by Amman et al. (2007).

The position of QTL qRFWs6.1 on chromosome 6 (4.58–4.61 Mbp) is similar to the position of q06_02 for leaf area reported by Frouin et al. (2018). The positions of 2 co-located QTLs qRLs6.1 and qRFWs6.3 on chromosome 6 (29.76–30.92 Mbp interval) are comparable to QTL #2098 for relative seminal root length reported by Prasad et al. (2000). Also, the location of QTLs qRLs8.1 on chromosome 8 (0.06–0.12 Mbp interval) co-locates to a08_04 for tiller number reported by Frouin et al. (2018), and the location of QTL qRLs8.2 on the same chromosome (2.33 Mbp) is comparable to QTL #2175 for leaf water content detected by Ul Haq et al. (2010). The positions of 2 QTLs qRLs8.3 and qRFWs8.1 on the same chromosome (at positions 21.51 and 26.73–26.94 Mbp) are similar to q08_05 and q08_06 for specific leaf area (SLA) reported by Frouin et al. (2018).

The location of qRFWs9.1 at 17.96 Mbp on chromosome 9 is comparable to QTL #2176 for leaf water content reported by Ul Haq et al. (2010) and to q09_05 for leaf area reported by Frouin et al. (2018), and the location of qRFWs11.1 at 21.68–21.98 Mbp interval on chromosome 11 is comparable to QTL #2178 for leaf water content reported by Ul Haq et al. (2010) and to q11_05 for leaf area reported by Frouin et al. (2018) and to qSKC11 for shoot Na^+^:K^+^ ratio detected by Naveed et al. (2018). The 2 QTLs including qRDWs12.1 and qRFWs12.1 on chromosome 12 (at 17.53 to 18.60 Mbp interval) locate to a similar region of QTLs #2168 for shoot Na^+^ concentration, #2173 for Na^+^:K^+^ ratio and #2174 for shoot dry weight (Ul Haq et al. 2010). Furthermore, the position of 2 co-located QTLs qSLs12.2 and qSFWs12.1 on this chromosome (21.08 Mbp) is comparable to q12_02 and q12_03 for root length reported by Frouin et al. (2018) and to qRL12 for root length reported by Naveed et al. (2018) (supplementary Table S11).

Based on comparison with locations of earlier reported QTLs, a complex genomic region on chromosome 1 at 32.33–32.54 interval, although it is adjacent to the QTL #2122 of Takehisa et al. (2004), it is adequately far from this QTL (~ 1.5 Mbp) to be distinguished as a different QTL, and hence it is a novel region containing 5 QTLs (qRDWs1.2, qRFWs1.3, qSLs1.1, qSFWs1.1, qTWs1.1) which was not, to our knowledge, reported before this study. Similarly, 5 other genomic regions detected in our GWAS signals are reported here for first that are associated to salinity-related traits: a genomic region at 35.26 Mbp on chromosome 2 (carrying qRDWs2.1 and qRFWs2.1), a genomic region at 20.04 Mbp on chromosome 6 (carrying qRFWs6.2), a genomic region at 6.12–7.32 Mbp interval on chromosome 10 (carrying qRLs10.1 and qRFWs10.1) and 2 genomic regions on chromosome 11 including 6.06 Mbp position (qRWCs11.1) and 24.96–25.85 Mbp interval (qRWCs11.2, qSFWs11.1, qSDWs11.1 and qTWs11.1).

In our study, the results for RDW and RFW were found more important than other traits, such that 7 and 15 QTL regions, respectively, tagged to 39 and 82 SNPs were identified on different chromosomes of rice. Out of these SNPs associated to RDW and RFW, 30 and 34 SNPs, respectively, located on chromosome 1 in a coordinate of 40.79–42.98 Mbp. This region carries 12 candidate genes (Table 11), some of them were not absolutely reported for salinity tolerance at seedling or any developmental stages in rice. More interesting genes are *Os01g0966000* and *Os01g0963000*. First gene (*Os01g0966000*) encodes a plasma membrane (PM) H^+^-ATPase (EC 3.6.1.3). It was shown that PM H^+^-ATPase family members play a well-known role in tolerance to abiotic stresses such as cold (Iswari and Palta 1989; Martz et al. 2006), drought (Du et al. 2015) and acid rain (Liang et al. 2015). Many studies also documented the importance of PM H^+^-ATPase in response to salinity stress in *Arabidopsis* and cereal plants such as wheat and rice (Ayala et al. 1997; Vitart et al. 2001; Gévaudant et al. 2007; Morgan et al. 2014; Chen et al. 2015a, b; Falhof et al. 2016). Second gene (*Os01g0963000*) encodes a peroxidase BP1 protein. The main role of this peroxidase such as other type I peroxidases is the removal and scavenging of reactive oxygen species (ROS) including H_2_O_2_ (Asada 1992; Shigeoka et al. 2002). The important role of type I peroxidases was well documented in plant stress tolerance (Koussevitzky et al. 2008; Dietz 2016). In a more recently work by using proteome assay it was revealed that peroxidase is a major category of proteins differentially regulated under salt stress at seedling stage between non-tolerant and tolerant rice genotypes (Lakra et al. 2019). The role of other genes in this complex region of chromosome 1 (40.79–42.98 Mbp) remains to be elucidated.

In addition to 2 above-mentioned genes, we identified three other important genes on chromosome 1 in our GWAS signals, including *Os01g0304100* (11.26 Mbp), *Os01g0624700* (24.95 Mbp) and *Os01g0812000* (34.51 Mbp). First gene resides in the vicinity of well-known *SalTol* QTL region and encodes a cation chloride cotransporter which was identified as a determinant of salt tolerance in previous works (Walia et al. 2005; reviewed in Waziri et al. 2016). Second gene (*Os01g0624700*) encodes a *WRKY* transcription factor (*WRKY 12*) which might play a role in salinity stress, but its precise mechanism of action remains to be determined. The involvement of WRKY family members in salinity tolerance were well documented (Hu et al. 2013; Li et al. 2013; Yan et al. 2014; Jiang et al. 2017). Third gene (*Os01g0812000*) encodes a transcriptional activator of gibberellin-dependent alpha-amylase expression (*GAMyb*) which is a unique gene of *GAmyb* class on chromosome 1 (see https://rapdb.dna.affrc.go.jp). Gibberlic acid (GA) has a known role in regulating α-amylase synthesis in aleurone cells via *GAmyb* which is necessary for expression of genes encoding α-amylase (Gubler et al. 1999). It was well documented that activation of *α*-amylases as major degradation factors of stored starches in the seed endosperm is needed for germination and post-germination growth (Lovegrove and Hooley 2000; Zou et al. 2008). Additional research has shown that *GAmyb* can transactivate other GA-regulated genes (Gubler et al. 1999; Cercós et al. 1999). Another *GAmyb* gene on chromosome 6 of rice at position 3.6 Mbp which was annotated as salt responsive DW40 3 (*SRDW3*), has a role in salt response (https://rapdb.dna.affrc.go.jp). This gene resides within two co-located QTL regions at normal condition (e. g qRDWn6.1 and qRFWn6.1) at a narrow coordinate on chromosome 6 (3.59–3.60 Mbp) (Tables 4 and 5). A DW40 type gene named *BnSDW1* was characterized in *Barssica napus* which causes salinity tolerance (Lee et al. 2010). Taking together, it seems that the *GAmyb* identified on chromosome 1 in this study maybe play a role in salt response.

A candidate gene on chromosome 2 (*Os02g0730300* at 30.4 Mbp) which is related to SL-R and RDW-R, encodes a high affinity potassium (K^+^) transporter (*HAK*). Potassium (K) affects all aspects of plant growth including resistance to pathogens and tolerance to abiotic stresses such as salinity, lodging, and drought (Ahmad et al. 2016). Limiting K loss supports osmotic adjustment, sustain cell expansion, ensures appropriate stomatal regulation and helps to sustain photosynthetic activity through photoassimilate translocation (Zörb et al. 2014), therefore, modulation of K transport is crucial under stress conditions. The putative function of the *KT/HAK/KUP* transporters has been predicted to play a key role in maintaining K homeostasis (Chen et al. 2015b; Li et al. 2017). Perception of osmotic stresses can trigger the transient K effluxes at the plasma membrane by impairing HAK5 activity (Brauer et al. 2016). *OsHAK1* overexpression plants exhibited higher K acquisition efficiency, a stronger growth phenotype and increased grain yield, especially when grown in adverse environmental conditions (Chen et al. 2017). Regarding to these findings, we suppose that our candidate gene *Os02g0730300* could play an inevitable role in response to salinity stress.

Another candidate gene on chromosome 6 encoding DnaJ domain protein C55 was identified in our GWAS signals. DnaJ as a heat shock protein has chaperon properties (Laufen et al. 1999; Miernyk 2001). A mitochondrial DnaJ protein (BIL2) in *Arabidopsis thaliana* confers growth and resistance against environmental stresses such as strong light and salinity stresses (Bekh-Ochir et al. 2013). More recently a few DnaJ genes were identified in rice that were up-regulated under salinity stress (Wang et al. 2015), all of which are different from our detected DnaJ (*Os06g0535300*). It seems that this gene is a novel DnaJ involved in salinity response. Another interesting candidate gene on chromosome 6 is *Os06g0717400* which encodes a pseudouridine synthase domain containing protein. Pseudouridines do not occur naturally in mRNA, but mRNA pseudouridylation is induced at specific sites under some stress conditions such as heat shock in yeast (Schwartz et al. 2014) and it was shown that the rate of translational read-through was increased after pseudouridylation of stop codon (Karijolich and Yu 2011). This finding indicates that mRNA pseudouridylation can influence gene expression at post-transcriptional level (Lovejoy et al. 2014). Interestingly, it was observed that, in spite of being constitutive RNA modifications, a few pseudouridines in yeast can be induced in small nuclear RNAs (snRNAs) under some stress conditions such as heat shock (Wu et al. 2011; van der Feltz et al. 2018). These unusual post-transcriptional RNA modifications can alter regulation of splicing patterns under stress conditions (Lovejoy et al. 2014; Karijolich et al. 2015). Based on these findings, the candidate gene *Os06g0717400* identified in our GWAS signals might play a role in salinity tolerance, but so far it was not reported the role of any pseudouridine synthase gene in stress tolerance in plants and hence, the mechanism of action of our candidate gene remains to be elucidated.

Six candidate genes were identified on chromosome 8, one of which was an ABI/VP1 transcription factor, ABI/VP1 TF (*Os08g0101000*). This gene was annotated as a gene involving in regulation of iron-deficiency response and tolerance in RAP-db (https://rapdb.dna.affrc.go.jp). A member of ABI/VP1 TF class in rice, ABI3/VP1 TF, is a IDE-binding TF and regulates expression of *IDEF1* via binding to RY element of its promoter under iron deficiency condition (Kobayashi et al. 2007). Since under salinity stress the absorption of mineral nutrients (including iron) by plant roots is inhibited due to excessive accumulation of Na^+^ ions, the root cells activate *IDE*- type genes by mediating ABI/VP1 TF to absorb higher amounts of iron and to overcome iron deficiency. Although *IDE*-type genes (such as *IDEF1* and *IDEF2* in rice) are constitutively expressed (irrespective of Fe level) which indicates their role in sensing Fe deficiency signals, it was suggested that *IDEF1* also plays an important role in activation of Fe deficiency-induced genes involved in Fe uptake under iron deficiency. Therefore, we suggest that ABI/VP1 TF gene identified in our research could play an indirect role in salinity tolerance via activating *IDE*-type genes which in turn transactivates the Fe deficiency-induced gene(s) to overcome iron deficiency by absorption higher amounts of Fe under salt stress. However, its essential role must be assessed experimentally via complementation tests such as genetic transformation and or RNAi. Another candidate gene on chromosome 8 is *Os08g0538700* which encodes a retinoblastoma-related protein (RBR). To date there is no direct evidence on the role of RBR in salt stress tolerance in plants. In an early research (Åmellem et al. 1996) it was suggested that human RBR suppresses cell growth under hypoxic stress. This protein responds to mitogenic and anti-proliferative signals to coordinate cell-cycle control with the cellular environment (Daria et al. 2008). A more recently research revealed that a retinoblastoma-binding protein of WD40 family plays an important role in response to salinity stress in brine shrimp (*Artemia sinica*). In plants, however, it was reported that RBR has a role in stem cell maintenance and cell differentiation in *Arabidopsis* roots (Wildwater et al. 2005; Borghi et al. 2010). A study by Ausín et al. (2004) has revealed the role of a retinoblastoma-associated protein named FVE in regulation of flowering time in *Arabidosis*. Also, it was reported that RBR is involved in regulating flowering time and cold response in *Arabidopsis* (Jeon and Kim 2011). A comparative transcriptome analysis in foxtail millet (*Setaria italica*) showed that a putative retinoblastoma-related protein was up-regulated during dehydration stress (Lata et al. 2010). In regard to common nature of responses to different environmental stresses and evidences on RBR role in some above-mentioned stresses, perhaps the RBR gene detected in current study has a putative role in salinity tolerance, mechanism of which needs to be elucidated under salinity stress.

Three candidate genes on chromosome 11 were identified in our GWAS signals. One of them is *Os11g0575600* which encodes a lipoxygense protein. The role of lipoxygenases in plant stress response was well documented (Babenko et al. 2017). *Arabidopsis Lipoxygenase* 1 (*AtLox1*) gene is involved in response to oxidative stress induced by cadmium exposure (Keunen et al. 2013). In pepper *CaLox1* plays a role in response to different environmental stresses including high salinity stress (Lim et al. 2015). Rice *Lox* genes, specifically *Lox2* are involved in response of rice to different environmental stresses including salinity stress (Rabbani et al. 2003; Walia et al. 2007; Roychoudhury et al. 2008; Liu et al. 2014; Islam et al. 2019). Another candidate gene on this chromosome (*Os11g0578700*) encodes an F-box protein. The F-box proteins are one of the largest class of functional proteins affecting diverse aspects of plant life. Involvement of F-box proteins in plant defense against abiotic stresses such as salinity stress was documented in previous researches (Jain et al. 2007; Zhang et al. 2008; Yan et al. 2011; Jia et al. 2017). More recently, Al-Tamimi et al. (2016) using a new association model identified an F-box encoding gene on chromosome 5 of rice for transpiration use efficiency (TUE) under saline condition. Accordingly, the candidate gene *Os11g0575600* identified in our research could play a role in salinity response, but its precise mechanism of action remains to be elucidated. In addition, within a complex region of chromosome 11 carrying 4 co-located QTLs viz. qRDWs11.2, qSFWs11.1, qSDWs11.1 and qTWs11.1 (24.96–25.85 Mbp interval) there are two putative candidate genes including *Os11g0644000* and *Os11g0648000*. *Os11g0644000* encodes a receptor-like cytoplasmic kinase (*RLCK 342*). It was reported that *OsSIK1*, a receptor-like kinase enhances rice tolerance to drought and salt stress (Ouyang et al. 2010), and also it was reported that the receptor-like kinase *OsSIT1* confers salt sensitivity in rice via regulating ethylene homeostasis (Li et al. 2014). Second gene (*Os11g0648000*) encodes a Na^+^/H^+^ antiporter annotated as *OsNHX2*. Four vacuolar Na^+^/H^+^ antiporters including *OsNHX2* were reported in rice (Bassil et al. 2012). Na^+^/H^+^ antiporters enhance the compartmentalization of Na^+^ into the vacuoles, and hence improve salt tolerance (Kumar et al. 2013; Amin et al. 2016), an issue which was validated by overexpression of *OsNHX1* in rice (Chen et al. 2007; Wang et al. 2012).

For validating the effect of identified candidate genes on salt tolerance, gene expression assays for 2 candidate genes (*Peroxidase BP1* and *PM H*^*+*^*-ATPase*) were conducted in tolerant and sensitive rice cultivars. Expression of *Peroxidase BP1* at early hours of salinity stress increased in both sensitive and tolerant cultivars, presumably to remove produced ROS, and then down-regulated, particularly in tolerant cultivar; in continue the expression of the gene was considerably increased at 72 h in both cultivars, but much more up-regulated in tolerant cultivar. In general, differential expression of peroxidases is one of factors involving in acquiring salinity tolerance in rice (Lakra et al. 2019). In the case of *PM H*^*+*^*-ATPase* also we observed similar expression pattern with minor differences. The gene was more up-regulated at 72 h after stress in tolerant cultivar, presumably to retain membrane function under salinity stress (Chen et al. 2015a, b; Falhof et al. 2016).

## Conclusion

The results for RDW and RFW were found more important than other traits, and known or novel candidate genes in this research can be used for improvement of salinity tolerance in molecular breeding programmes. Further study and identification of effective genes on salinity tolerance by the use of candidate gene-association analysis can help to precisely uncover the mechanisms of salinity tolerance at molecular level. A time dependent relationship between salt tolerance and expression level of genes (e. g. *PM H*^*+*^*-ATPase*, and *Peroxidase BP1*) could be recognized.

## Methods

### Plant Materials and the Growth Conditions

One hundred fifty five varieties of rice (Table S1) were randomly selected from a larger panel of rice varieties (from the Chang Genetic Resources Center, International Rice Research Institute (IRRI), Philippines). The experiment was carried out in Shahid Beheshti University, Tehran, Iran. These accessions were cultured in early April (2017) in three replications under 2 environments including normal and salt stress conditions. Plant materials were evaluated in a factorial experiment (155 genotypes and two levels of salt stress induced by NaCl). To do this, seeds of each variety were surface-sterilized with 10% hypochlorite sodium, washed 3 times using distilled water and put in paper towels for germination in a germinator with 28 °C. After germination they were transferred to growth chamber with 25 °C, 60% relative humidity and 16/8 h light/dark regime.

### Salinity Treatment and Phenotypic Evaluations

Phenotypic evaluations were conducted in a completely randomized design (CRD) with three replicates at controlled condition in a growth chamber. The control treatment (normal environment, N) included Yoshida solution, whereas the salinity stress treatment (100 mM NaCl) was induced by adding needed volume of 1 M NaCl to the Yoshida solution. The Yoshida solution included macro and micro-elements. During the growth of the seedlings, the solution pH was set to 5.5 and the medium salinity was set to the desired level every day. Also, every 3 days the solution was renewed.

In order to evaluate the given traits after normal and salt treatments (14 days after transfer to growth chamber), ten seedlings from each replicate were randomly selected and separated to root and shoot sections. The growth of seedling sections including root length (RL), shoot length (SL), root fresh weight (RFW), shoot fresh weight (SFW), was measured. Furthermore, fresh roots and shoots were oven-dried at 80 °C for 24 h. After that, root dry weight (RDW) and shoot dry weight (SDW) were also measured. Turgid weight (TW) was measured 24 h after floating the shoots in distilled water. The oven-dried seedling shoots were weighted to calculate relative water content (RWC). Also, some salinity-relative traits were calculated including SL-R, RL-R, RFW-R and RDW-R using appropriate formulae (for example SL-R = SL_salt_/SL_normal_). To determine differences among genotypes (G), salt treatment levels (T) and G × T interactions, the phenotypic data were analyzed by using general linear model (GLM) in SPSS v.19 software (Gray and Kinnear 2012). Differences between means were compared using LSD test at the 5% level of significance (*p* ≤ 0.5).

### Genome-Wide Association Study (GWAS)

The genotypes of all rice varieties were determined at 37867 SNP loci which were downloaded from Gramene database (www.gramene.org). By the use of TASSEL software, these SNPs were filtered for monomorphic loci and loci with minor allele frequency (MAF) below 5%. In order to determine the number of the real subgroups (K) in the studied rice collection, the STRUCTURE software was used (Pritchard et al. 2000). The number of the initially assumed subpopulations (K) was considered to be between 1 and 10; and in order to increase the accuracy of the estimation of the parameters, 10 independent replicates were specified for each K subpopulation. Finally, the optimized number of the subpopulations (K) was estimated on the basis of the ΔK method according to Evanno et al. (2005). After determining the number of real subpopulations, the respective Q matrix was extracted to be used in the association mapping stage.

In most of the GWAS studies of plants, r^2^ is used for estimating the extent of linkage disequilibrium (LD) because it presents all of the information related to linkage between the marker and the QTL (Flint-Garcia et al. 2003, Remington et al. 2001; Courtois et al. 2013). r^2^ is estimated to be between 0.1 and 0.2 which is the minimum threshold for a significant association between the SNP marker and given traits. The association analysis was done by the use of SNP markers whose allele frequencies were higher than 0.05. The analysis was done through the MLM method by the use of TASSEL software (Bradbury et al. 2007). In the MLM method, in order to prevent the false marker-trait associations, in addition to Q matrix (coefficients of population structure), K matrix (the kinship coefficient of the varieties) was also used. Thus the used model was MLM-Q-K. In this method, the genotypic effects (SNP markers) and the coefficients of the population structure (Q) are taken as the fixed effects, and the varieties’ kinship coefficients (K) are taken as the random effects (Bradbury et al. 2007). MLM Model is as following:

$$ \mathrm{P}=\upmu +\mathrm{M}+\mathrm{Q}+\mathrm{K}+\mathrm{E} $$

Where P is the phenotype, M and Q are the genotypic fixed effects and population structure, respectively, and K is the effect of random kinship of the samples, and E is the residual effects. The association analysis was done separately for each condition, so that the mean value of replicates for each trait was used as the phenotypic data. After association analysis, the GWAS results were presented as Manhattan plots based on the negative log_10_ transformed observed *p*-values for each SNP-trait association. We used a threshold of 1e-04 to declare a SNP significant (Kaler et al. 2017).

### Identification of the Candidate Genes and Haplotype Analysis

In order to identify the genes underlying the QTLs of salt tolerance, overlapping of the physical genomic regions of these QTLs (=their associated SNPs) and any gene deposited on the Rice Annotation Project database, RAP-db (https://rapdb.dna.affrc.go.jp), was assessed. The genes annotated as hypothetical, non-protein coding and transposable element were discarded. In addition, higher sensitivity was put on stress-related genes whose sequence coincided to associated SNPs. Then, from the gene lists, the candidate genes were selected based on the predicted function (biological processes) in relation with the trait of interest.

In haplotype analysis genomic blocks on each chromosome were scanned in adjacent regions using SNP data of candidate genes in our rice accession pannel. Block haplotypes with frequency lower than 5% were omitted.

### Validation Assays

In the present study, the expression pattern of several new genes involved in salt tolerance of rice was investigated by real-time PCR in two sensitive (ARC6578 and Shoemed) and one tolerant (Bombilla) rice accessions that were selected based on previous work (Nayyeripasand et al. 2019). RNA samples were extracted from 20-day-old seedlings treated with NaCl (100 mM) at three times after salt stress (24, 48 and 72 h). Gene expression analysis was performed on 2 candidate genes (including *Proxidase BP1 precursor* and *Plasma Membrane H*^*+*^*-ATPase*) using real-time PCR. The Actin gene was used as the reference gene in this assay. Furthermore, a co-expression assay was conducted using riceFREND database (Sato et al. 2013) (https://ricefrend.dna.affrc.go.jp/) for all candidate genes.

## Supplementary information

**Additional file 1: Table S1.** Rice varieties used in this study. **Table S2.** Analysis of variance for the studied traits in germplasm set of rice under normal and salinity stress conditions. **Table S3.** Pearson correlation coefficients between different traits under normal condition. **Table S4.** Pearson correlation coefficients between different traits under salinity condition. **Table S5.** Co-ancestory coefficients (Q) in 4 sub-populations for germplasm set used in the study. **Table S6.** Results of association mapping using MLM-Q-K model for detection of SNPs with significant association to the studied traits under normal condition. **Table S7.** Results of association mapping using MLM-Q-K model for detection of SNPs with significant association to the studied traits under salinity condition. **Table S8.** Approximate expression levels of candidate genes (in view of transcript per million, TPM) inferred from EST sources in Unigene database. **Table S9.** Haplotype analysis of candidate genes on rice chromosomes. **Table S10.** Co-expression analysis of candidate genes using riceFREND database. **Table S11.** Comparison of the locations of the detected genomic regions in this study (QTLs) and the known reported QTLs.

**Additional file 2: Figure S1.** a. A histogram showing the frequency distribution of 8 traits phenotyped for germplasm set used in the study under control condition. b. A histogram showing the frequency distribution of 8 traits phenotyped for germplasm set used in the study under salinity condition. **Figure S2** Results of clustering the entire population. (A) Determining the optimized number of K clusters based on Evanno et al. (2005) method. (B) Bar plot of the clustering result showing the population structure derived from the STRUCTURE software. **Figure S3.** Manhattan plots of p-values analyzed using mixed linear model (MLM) controlled for population structure and kinship of rice genotypes for derived traits under salinity condition.

## Data Availability

All relevant data have been provided as Tables, Figures with in the text and in the following supplementary data.

## Abbreviations

ANOVA: Analysis of variance
GWAS: Genome-wide association study
LD: Linkage disequilibrium
MLM: Mixed linear model
PM: Plasma membrane
QTL: Quantitative trait loci
RDW: Root dry weight
RFW: Root fresh weight
RL: Root length
RWC: Relative water content
SDW: Shoot dry weight
SFW: Shoot fresh weight
SL: Shoot length
SNP: Single nucleotide polymorphism
TW: Turgid weight
