# Supplementary material for: Genome-Wide Association Study (GWAS) to Identify Salt-Tolerance QTLs Carrying Novel Candidate Genes in Rice During Early Vegetative Stage
Source: Rice (N Y). 2021 Jan 9;14:9. doi: 10.1186/s12284-020-00433-0 (PMC7797017; doi:10.1186/s12284-020-00433-0)
Supplement: Supplementary file 2 — Additional file 2: Figure S1. a. A histogram showing the frequency distribution of 8 traits phenotyped for germplasm set used in the study under control condition. b. A histogram showing the frequency distribution of 8 traits phenotyped for germplasm set used in the study under salinity condition. Figure S2 Results of clustering the entire population. (A) Determining the optimized number of K clusters based on Evanno et al. (2005) method. (B) Bar plot of the clustering result showing the population structure derived from the STRUCTURE software. Figure S3. Manhattan plots of p-values analyzed using mixed linear model (MLM) controlled for population structure and kinship of rice genotypes for derived traits under salinity condition. [file 12284_2020_433_MOESM2_ESM.docx]

**Supporting information**


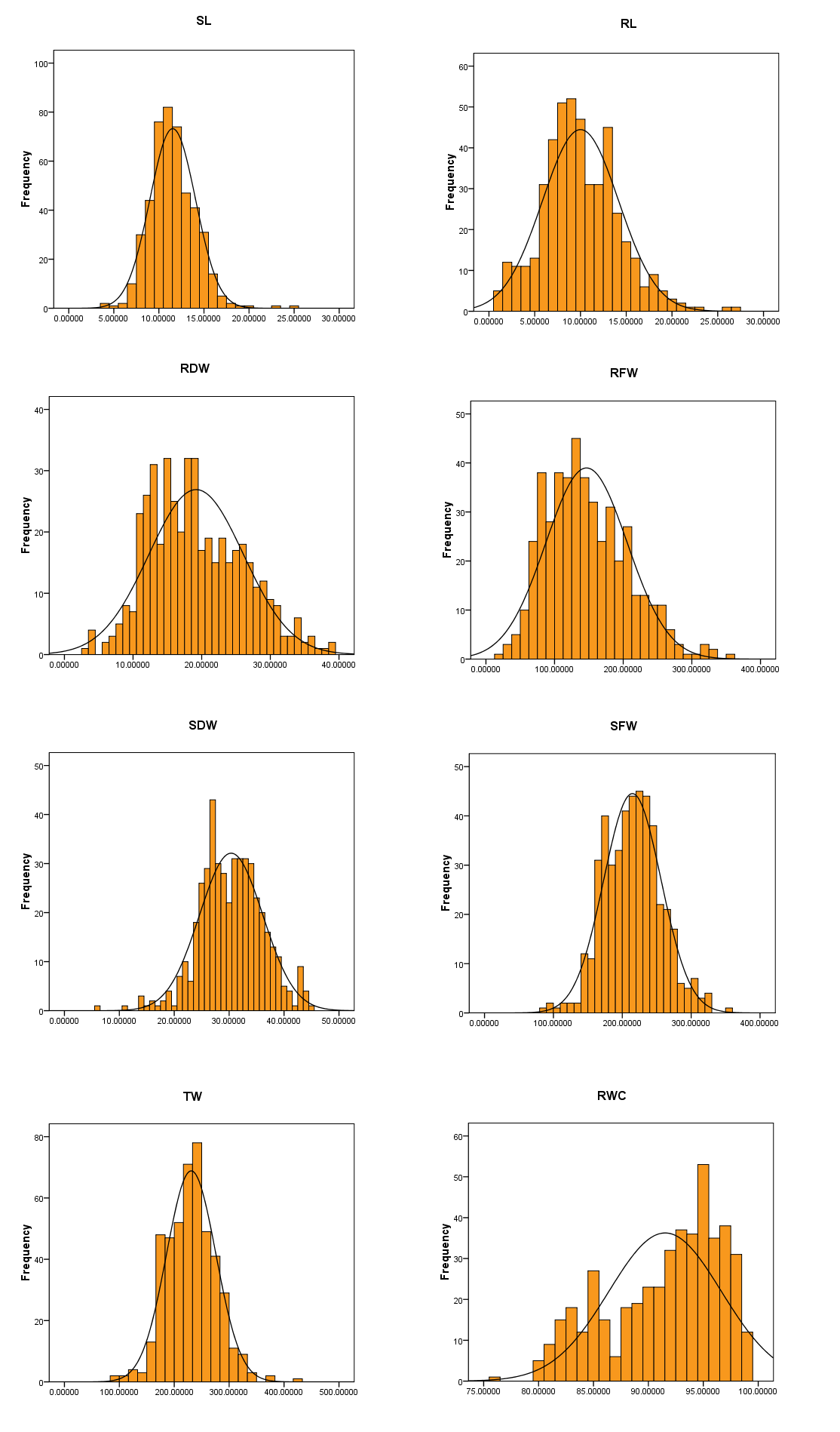


**Figure S1a.** A histogram showing the frequency distribution of 8 traits phenotyped for germplasm set used in the study under control condition.


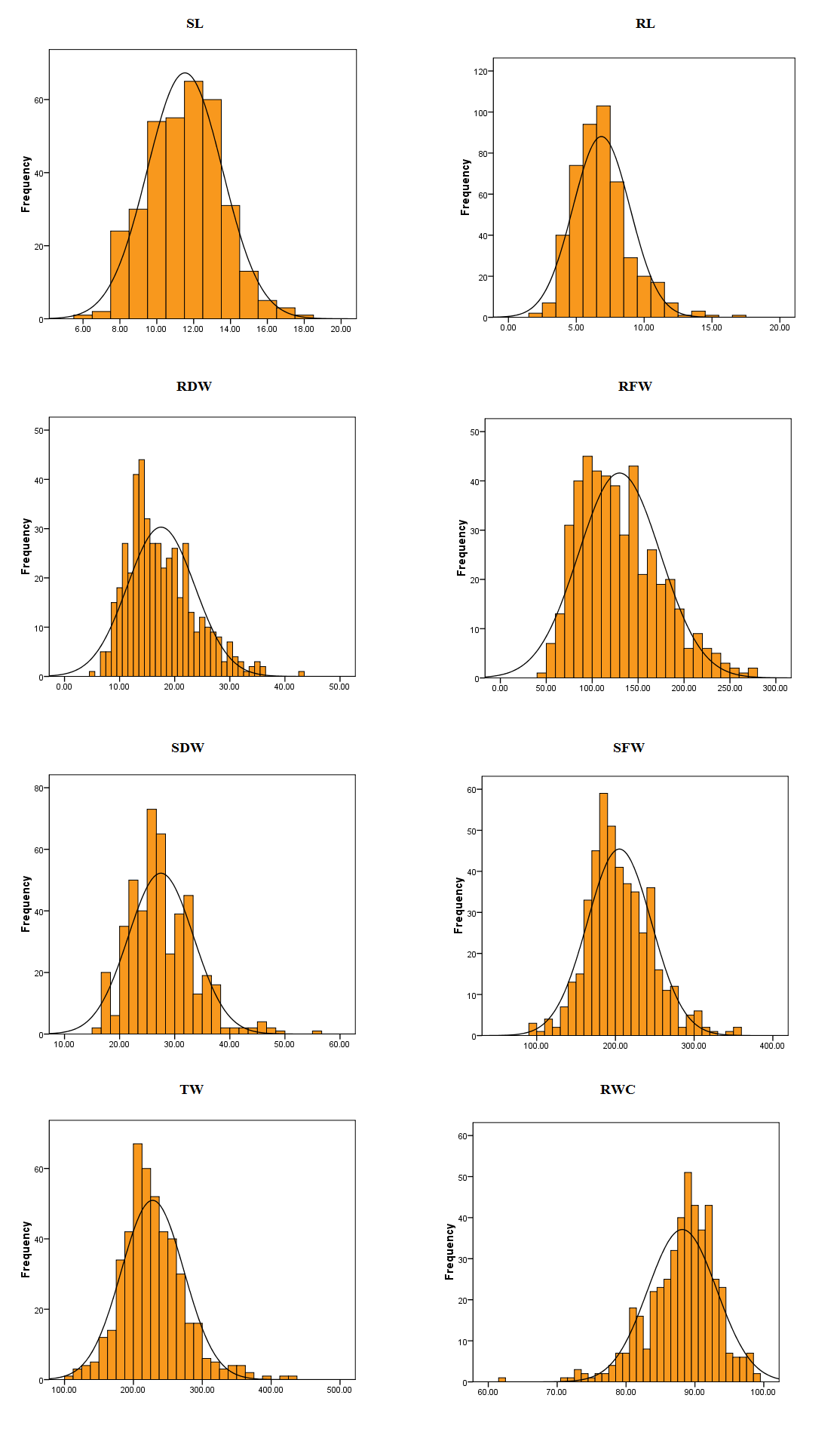


**Figure S1b.** A histogram showing the frequency distribution of 8 traits phenotyped for germplasm set used in the study under salinity condition.

**
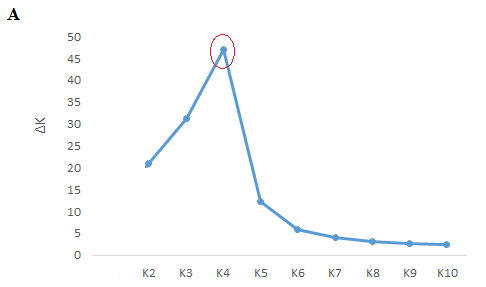
**

**
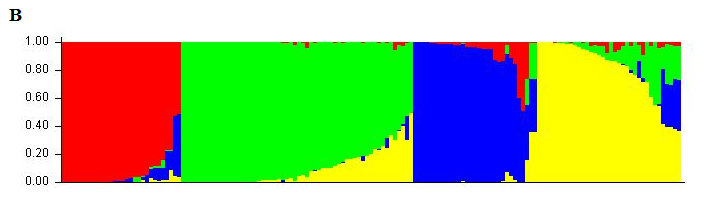
**

**Figure S2** Results of clustering the entire population. (A) Determining the optimized number of K clusters based on Evanno et al (2005) method. (B) Bar plot of the clustering result showing the population structure derived from the STRUCTURE software.


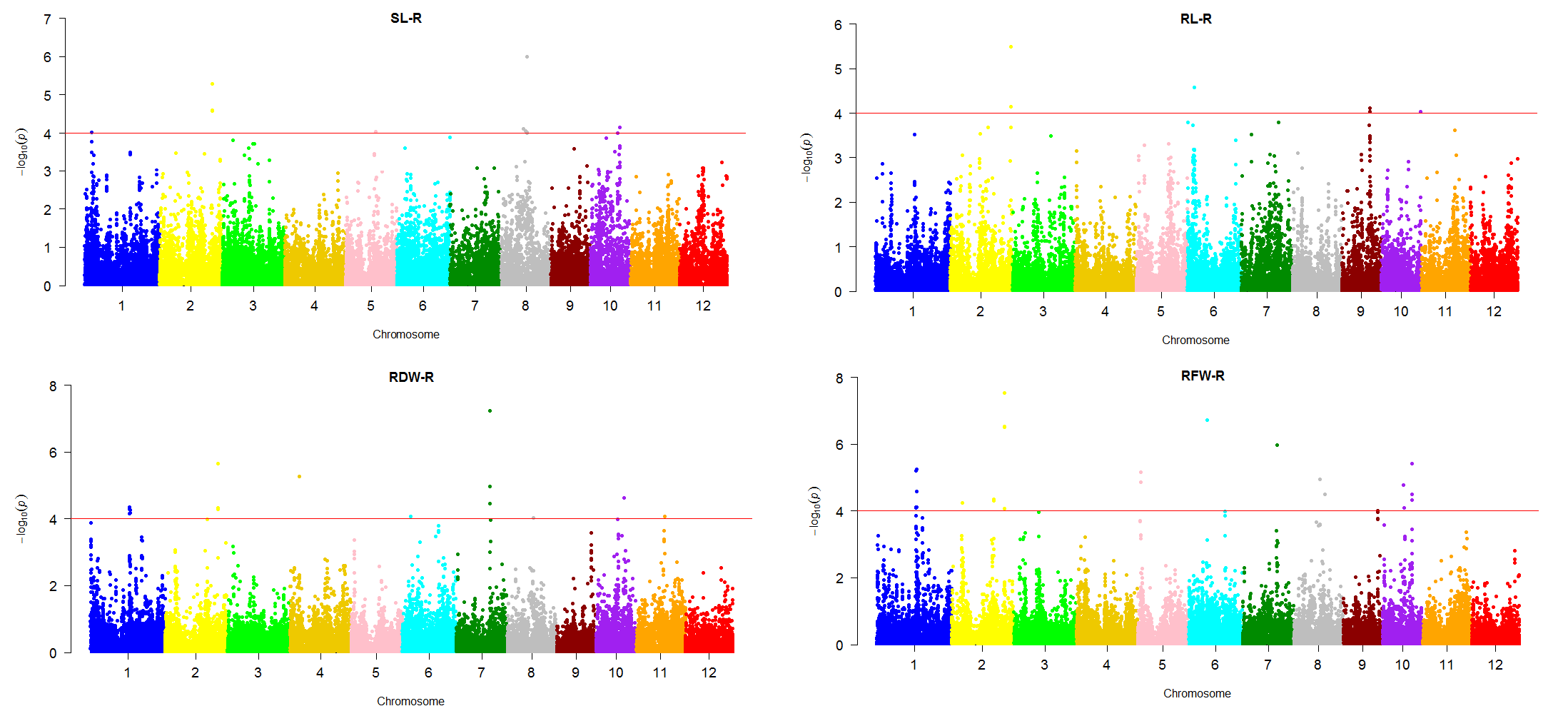


**Figure S3**. Manhattan plots of p-values analyzed using mixed linear model (MLM) controlled for population structure and kinship of rice genotypes for derived traits under salinity condition.
